# Femtosecond laser-assisted removal of an intracorneal chestnut, a case report

**DOI:** 10.1186/s12886-018-0875-2

**Published:** 2018-08-28

**Authors:** Yong Jie Qin, Jin Zeng, Hong Liang Lin, Wen Juan Xie, Yan Zhang, Hai Ke Guo, Hong Yang Zhang

**Affiliations:** 1 Department of Ophthalmology, Guangdong Eye Institute, Guangdong General Hospital and Guangdong Academy of Medical Sciences, 106 Zhongshan Er Road, Guangzhou, 510080 China; 2 Shanghai Heping Eye Hospital, 61 Shanghai Yiminhe Road, Shanghai, China

**Keywords:** Femtosecond laser, IEK, Corneal injury, Intracorneal foreign body, Chestnut

## Abstract

**Background:**

To report a case of femtosecond laser-assisted removal of an intracorneal chestnut.

**Case presentation:**

A chestnut was obliquely protruding to the stroma of cornea and it was localized at the paracentral region on the left eye of a 32-year-old man. The best-corrected visual acuity (BCVA, in decimal values) was 0.6 in the injured eye. The white ulcers with feathery edges or satellite infiltrates were not observed in the lesion, and the anterior chamber was deep and quiet. Anterior segment optical coherence tomography (AS-OCT) demonstrated that the original entry path of the foreign body had been sealed, spanning a thickness of approximate 152 μm. In view of location of the intraocular chestnut at the paracentral region, femtosecond laser was applied according to the procedures of IntraLase Enabled Keratoplasty (IEK) to create an anterior lamellar flap rapidly and precisely. The lamellar flap was easily separated with a flap lifter, and the chestnut was removed entirely using a pair of forceps. In 3 days after surgery, the patient complained of mild pain and blurred vision. These symptoms were relieved after treatment with the eyedrops. At three-month follow-up, the corneal wound was healed well, and the BCVA was greatly improved to 1.2 in the left eye. A dot-like haze was observed corresponding to the scar at the site of foreign body removal. No surgical induction of corneal astigmatism was found in the corneal topography.

**Conclusions:**

Without induction of a visually significant scar and corneal astigmatism, the IEK procedure of femtosecond laser is of particular interest as it provides a unique method for removal of intracorneal foreign bodies impinging on the visual axis.

## Background

Corneal foreign bodies are a common type of eye injury involving the foreign substances, such as metals, glasses or organic materials that superficially adhere to or embed in the cornea. The well tolerated substances without precipitating a pathological reaction to cornea can be left in the cornea and followed up regularly [1]. Some objects that are poorly tolerated by the patients must be promptly removed. Surgeries are suggested to remove the substance that is localized at the paracentral region and causing vision loss [2]. However, aggressive attempts to remove deep fragments may cause a visually significant scar and result in undesirable distortion of the corneal topography [3]. Femtosecond laser is an accurate technology to make extremely smooth and precise cuts. It has been commonly used in corneal refractive surgery [4]. Here we performed a reliable procedure using femtosecond laser to remove an intracorneal chestnut.

## Case presentation

**Fig. 1:**
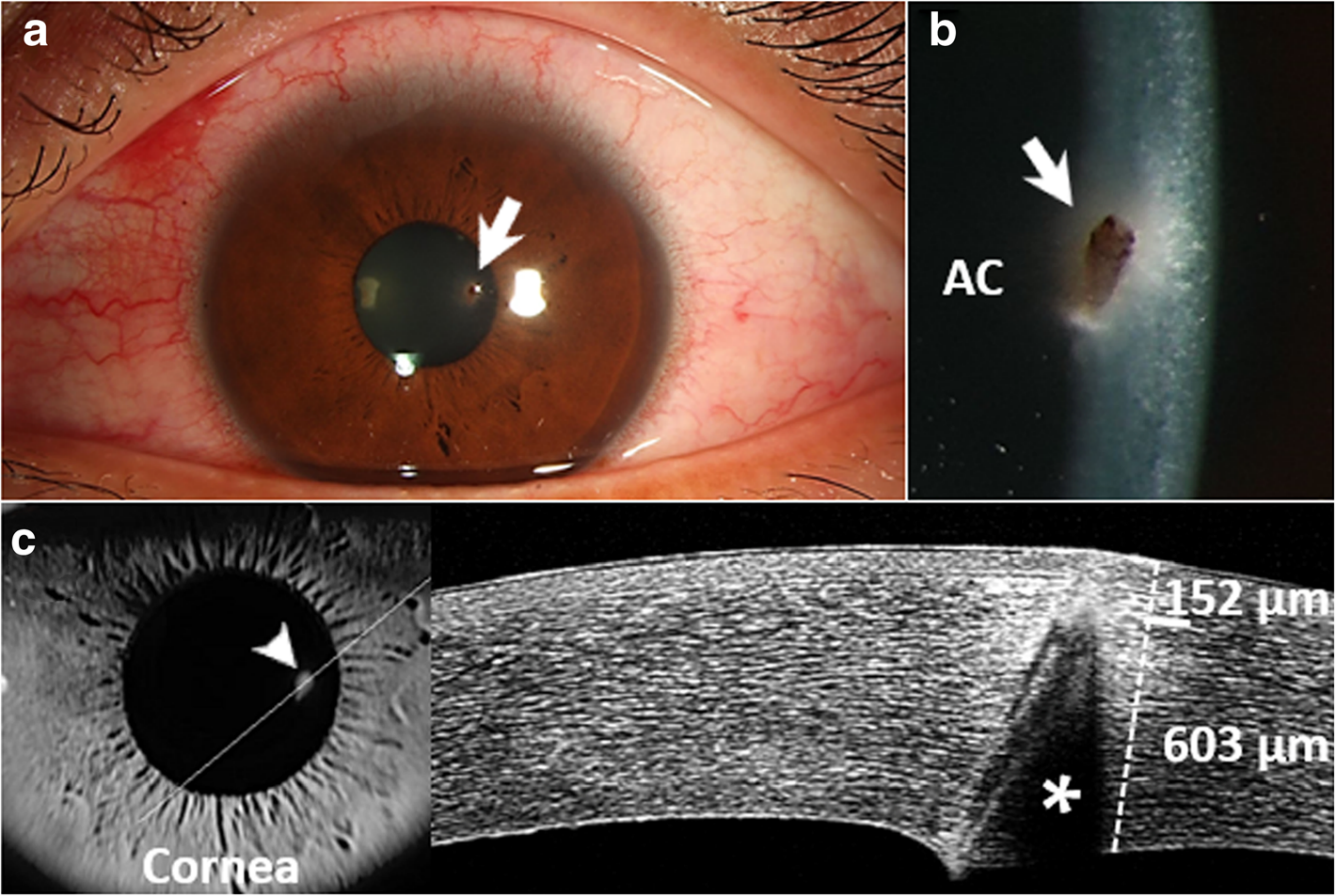
**a** Slit-lamp examination demonstrated a chestnut embedded in the corneal stroma (arrow). **b** The chestnut was obliquely protruding to Descemet membrane but not penetrating to the anterior chamber (arrow). **c** The white line through the chestnut (arrowhead) indicated the position of AS-OCT imaging probe. The shadow (asterisk) shown in AS-OCT corresponded to the location of the chestnut. Analysis with caliper tool showed an approximate 152 μm thickness of sealed tissue at the original entry path of the chestnut. AC, anterior chamber

A 32-year-old man presented with the symptoms of foreign body sensation and blurred vision in the left eye 3 days before presentation. The best-corrected visual acuity (BCVA, in decimal values) was 1.2 in the right eye and 0.6 in the left eye. Slit-lamp biomicroscopy (BX-900, Haag-Streit AG, Koeniz, Switzerland) of the left eye revealed an intracorneal foreign body, localized at the paracentral region, obliquely protruding to Descemet membrane with no penetration into the anterior chamber (Fig. 1). The original entry path of the foreign body had sealed and epithelialized, leaving a sub-epithelial opacity and edematous stroma (Fig. 1b and c). The shadow effect shown in the anterior segment optical coherence tomography (AS-OCT, RTVue XR, Optovue, Inc., Fremont, CA) corresponded to the location of the intracorneal chestnut (Fig. 1c). The corneal thickness was approximate 755 μm at the site of lesion, of which 152 μm distance from the sealed corneal epithelium to the chestnut (Fig. 1c). The white ulcers with feathery edges or satellite infiltrates were not observed. The intraocular pressure, anterior chamber, lenses and the fundi appeared normal. No signs of systemic disorders were found in the presented case.

**Fig. 2:**
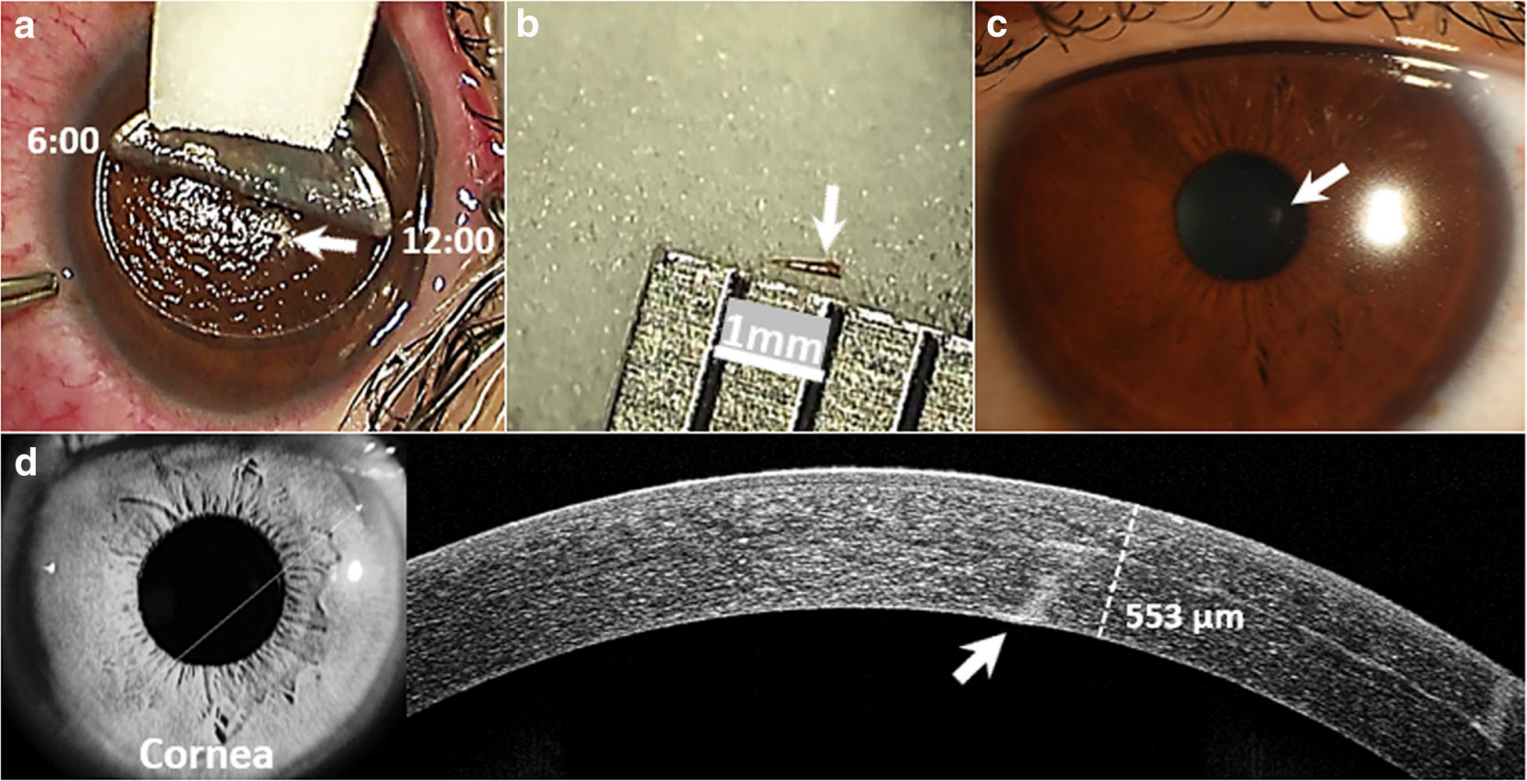
**a** Anterior lamellar flap of the cornea was created from 12:00 to 6:00 in the left eye to expose the chestnut (arrow). **b** A chestnut, around 600 μm in diameter, was removed completely (arrow). **c** Three months after surgery, a dot-like haze was noted at the site of the chestnut removal (arrow). **d** The white line indicated the position of AS-OCT assessment. A hyper-reflectivity illustrated in AS-OCT was corresponded to the haze that caused by the chestnut (arrow). The corneal thickness was 553 μm at three-month follow-up

This study conformed to the principles of the Declaration of Helsinki and was approved by the Institutional Ethics Committee of Guangdong General Hospital and Guangdong Academy of Medical Sciences. After discussing with the patient and informed consent was obtained, femtosecond laser was applied using the protocols of IntraLase Enabled Keratoplasty (IEK, iFS™ Advanced Femtosecond Laser System). Following parameters were used: 300 μm lamellar depth, 7.5 mm diameter, 1.20 μJ energy, and cut angle with 180 degrees from 12 o’clock to 6 o’clock in the left eye (Fig. 2a). Under strict aseptic precautions, IEK was performed to create an anterior lamellar flap according to the routine procedures. The lamellar flap was easily separated with a flap lifter to expose the superior side of the chestnut. As shown in Fig. 2a, the chestnut was then removed entirely with a pair of forceps under a surgical microscope (OPMI LUMERA 700, Carl Zeiss Meditec, Jena, Germany). After removal of the chestnut, no fluorescein leakage was found with the Seidel test and the wound was washed with 250 mL normal saline containing 40 mg gentamycin. The edges were dried for 3 min using a surgical sponge, and a soft contact lens (Extended wear, PureVision. Bausch and Lomb, NY) was applied over the surface. No suture was applied. The chestnut, shown in Fig. 2b**,** was inoculated onto Sabouraud glucose agar and chocolate agar to detect potential growth of fungi and bacteria.

**Fig. 3:**
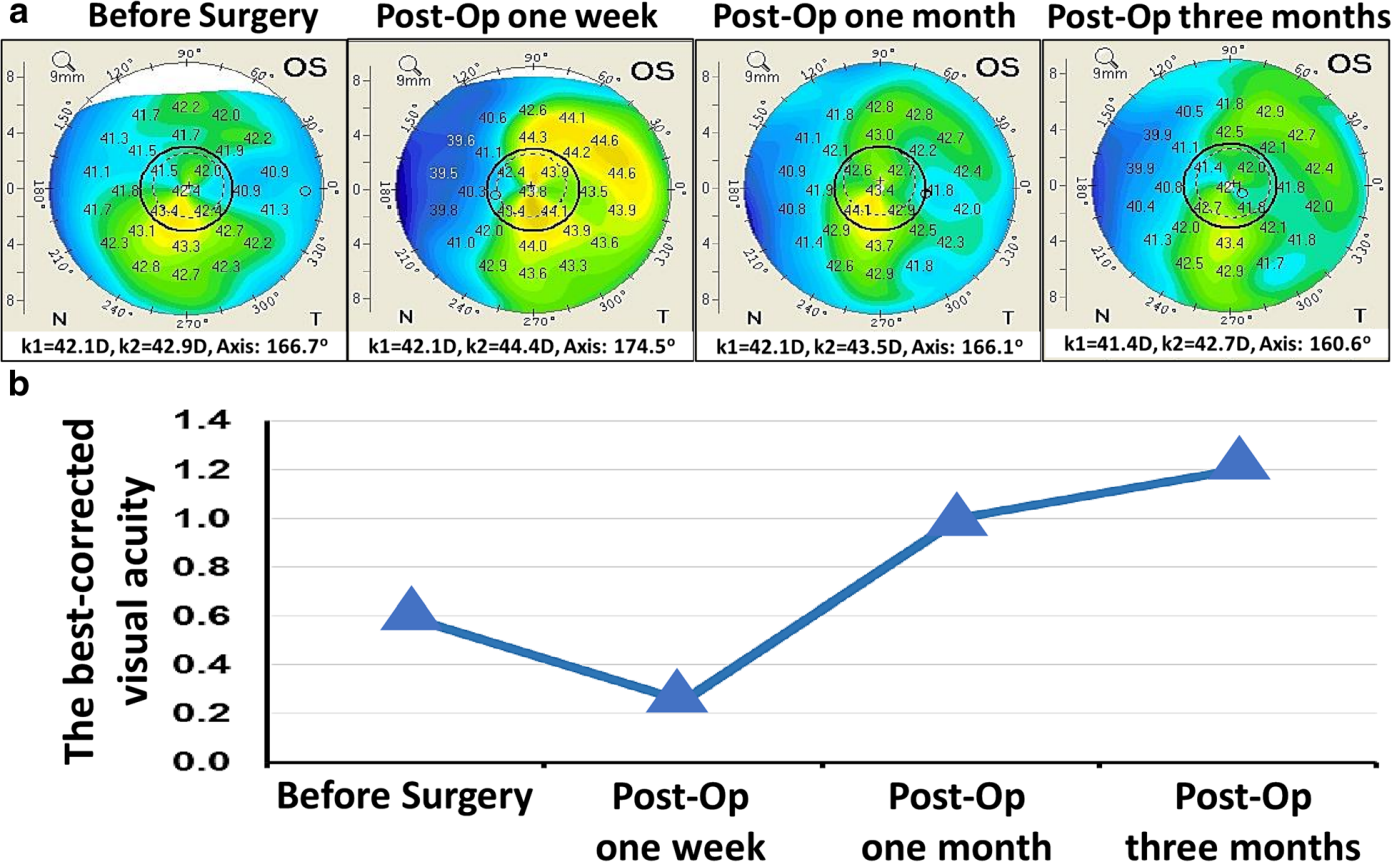
**a** Refractive corneal topography of the left eye (OS) was taken before surgery (K_1_ = 42.1D, K_2_ = 42.9D, Axis: 166.7^o^), Post-Op 1 week (K_1_ = 42.1D, K_2_ = 44.4D, Axis: 174.5^o^), Post-Op 1 month (K_1_ = 42.1D, K_2_ = 43.5D, Axis: 166.1^o^), and Post-Op 3 months (K_1_ = 41.4D, K_2_ = 42.7D, Axis: 160.6^o^). **b** The decimal BCVA of the left eye was improved gradually to 1.2 at the last follow-up

Before the surgery, Levofloxacin (Santen Pharmaceutical Co., Ltd. Japan) was prescribed for four times per day, and postoperatively, TobraDex (tobramycin 0.3% and dexamethasone 0.1%, s.a. Alcon-Couvreur n.v. Puurs, Belgium) was included and tapered weekly over a month. In 3 days after surgery, the patient complained of mild pain and blurred vision. These symptoms were relieved after treatment with the eyedrops. At three-month follow-up, a dot-like haze was noted in the cornea, which was corresponded to the scarring formation at the site of foreign body removal (Fig. 2c and d). As shown in Fig. 3, assessment with corneal topography (Oculus Pentacam Typ 70,700, Topcon, Tokyo, Japan) demonstrated that there was no surgical induction of corneal astigmatism compared to the preoperative astigmatism (Pre-Op, K_1_ = 42.1D, K_2_ = 42.9D, Axis: 166.7^o^ vs 3-month Post-Op, K_1_ = 41.4D, K_2_ = 42.7D, Axis: 160.6^o^), and the Post-Op decimal BCVA in the left eye was improved gradually from 0.3 to 1.2. The results in microbiological culture of fungi and bacteria were negative.

## Discussion and conclusion

Conventionally, removal of the intracorneal foreign bodies was manipulated with a scalpel blade and a needle. This technique required closing the peripheral lamellar pocket with suture, which may induce a visually significant scar [5]. A concern in this case was that the BCVA of the injured eye was still good enough and the chestnut embedded in the stroma was localized at the paracentral region. Inappropriate manipulations to dislodge it could induce visual impairment. A simple and effective technique was introduced to remove the corneal foreign body with a suture needle [6]. But it was considered unsuitable in our case because the original entry path of the chestnut had been sealed. Femtosecond laser generated sutureless anterior lamellar incisions, and improved the vision with rapid visual rehabilitation and no significant astigmatism induction [4, 7]. It was recently reported to remove a corneal scar caused by an impacted stromal glass [8]. Therefore, femtosecond laser was adopted in our case to create an anterior corneal flap. The desired depth and cut angle allowed us to remove the chestnut effectively. Of note, his BCVA was greatly improved, and the large and deep lamellar incision did not change the refractive error and did not induce scaring formation. Moreover, the chestnut was removed entirely, and no fluorescein leakage was observed in the wound, supporting that the procedure was extremely safe without disrupting the chestnut and causing penetration of the foreign body in ocular tissues.

The AS-OCT imaging demonstrated that the entry wound of the chestnut had been sealed with approximate 152 μm thickness, indicating that the lamellar-cut depth should be greater than that in order to grasp the chestnut. We thereby created an anterior lamellar flap with 300 μm depth to expose the free superior edge of the chestnut. Our results demonstrated that the chestnut can be simply removed with a pair of forceps. Thus, AS-OCT is a valuable tool for management of a corneal foreign body.

The concern with the use of femtosecond laser to remove the chestnut was the spreading of infections. The mature chestnut contains clusters of sharp and yellow-brown thorns that might harbor filamentous fungi leading to fungal keratitis [9]. In this case, the fungal infection was excluded. Because no sign of infections was noted, the cornea and the anterior chamber seemed to be quiet after injury, and postoperatively, the corneal wound was healed well, and no causative agent was found in the microbiological investigation. However, a careful examination is suggested to exclude fungal and bacterial infection before the surgery.

In conclusion, the femtosecond laser-assisted IEK is a precise procedure to create a lamellar flap without distorting the corneal topography. Our results suggest that femtosecond femtolaser-assisted IEK is a promising technique for the removal of intracorneal foreign bodies, particularly for those embedded in the paracentral region.

## Abbreviations

AC: Anterior chamber
AS-OCT: Anterior segment optical coherence tomography
BCVA: Best-corrected visual acuity
IEK: IntraLase Enabled Keratoplasty
Post-Op: Post-operative
